# Effect of Freeze-Drying on the Engineering, Cooking, and Functional Properties of Chickpea Cultivars Grown in an Indian Temperate Climate

**DOI:** 10.3390/foods14101687

**Published:** 2025-05-10

**Authors:** Najeebah Farooq, Idrees Ahmed Wani

**Affiliations:** Department of Food Science & Technology, University of Kashmir, Srinagar 190006, India; najeebah18@gmail.com

**Keywords:** chickpeas, freeze drying, cooking properties, hard-to-cook defect

## Abstract

The present study investigates the impact of freeze drying on the physical, cooking, and functional properties of some chickpea (*Cicer arietinum*) cultivars. Freeze drying was applied to reduce the cooking time in addition to evaluating its effect on other quality attributes. The results revealed a significant reduction in cooking time in the freeze-dried chickpeas (67.00–77.33 min) compared to the control chickpeas (80.33–93.66). Additionally, functional properties were enhanced, such as the water absorption capacity, which increased from 0.84–0.98 g/g to 1.051–1.24 g/g, the oil absorption capacity, which increased from 0.73–0.98 g/g to 0.909–980 g/g, and the foaming capacity, which increased from 42.58–45.16% to 44.37–47.20%. The textural analysis revealed that freeze drying resulted in a decrease in hardness from 2.72–3.91 kg to 1.48–2.05 kg among the cultivars. The structural analysis indicated notable modifications in starch granules, supporting the observed changes in pasting behavior, which exhibited increased peak and breakdown viscosities. However, a reduction in antioxidant activity, viz., DPPH, TPC, TFC, and FRAP, was observed, indicating a potential trade-off between the preservation technique and nutritional quality. This study underscores the potential of freeze drying to improve the cooking and functional properties of chickpeas, with a special focus on their cooking properties.

## 1. Introduction

Pulse grains are an affordable source of protein for human diets, typically comprising 18–25% of their weight. Among these, chickpeas (*Cicer arietinum* L.) are widely used as a protein alternative, especially for individuals following vegetarian or vegan diets. Around 18.1 million metric tons of chickpeas were produced globally in 2022 according to the latest complete statistics available. With over 13.5 million metric tons, India was the top producer. Australia came in second with over 876.5 thousand metric tons, as per [1]. Chickpeas are the third most produced grain legume, and they are consumed in countries such as China, Pakistan, India, Mexico, Turkey, Ethiopia, and the USA. Chickpeas are rich in various important micro- and macronutrients, such as carbohydrates (60–65%), crude proteins (18.4–29.0%), dietary fiber (4–6%), and fat (4.5–6.6%). Starch is the prominent carbohydrate in chickpeas. In addition to this, chickpeas also contain various organic compounds, such as polyphenols and isoflavones, which reduce the risk of various diseases like diabetes mellitus, inflammation, metabolic syndrome, and hypertension. Chickpeas also contain certain antinutritional factors like hemagglutinins, trypsin inhibitors, phytic acid, saponins, and tannins that decrease their digestibility, nutrient value, and sensory quality. This problem can be avoided by using appropriate processing methods like physical treatments (milling and soaking), biochemical treatments (germination and fermentation), and thermal treatments (roasting and extrusion). Using tools like scalpers, air screens, and gravity-based separators, chickpeas are first cleaned and graded to get rid of contaminants and separate the seeds according to size, weight, and color. Pre-milling procedures, which include wet (like soaking and red earth mixing) and dry (like pitting, tempering, and drying) methods, as well as more sophisticated ones like enzymatic, hydrothermal, ultrasonic, and microwave treatments, are essential for removing the seed coat. Depending on the kind of chickpea (Desi vs. Kabuli), soaking weakens the link between the seed coat and cotyledons, which makes dehulling and splitting easier. In addition to preparing the seed for easy dehulling, roasting is a popular thermal approach that employs methods including sand, pan, oven, microwave, and continuous tumble roasting. Each of these methods has unique heat-transfer mechanisms that affect texture, water loss, and nutrient retention. Using tools like hammers, discs, stones, or roller mills, milling turns entire seeds or cotyledons into flour. The qualities of the flour and damage to starch are affected by the size of the particles and the speed at which the milling occurs. Chickpea flour can be fractionated using either dry or wet methods to produce enriched protein, starch, and fiber fractions. While wet fractionation (such as alkaline extraction, isoelectric precipitation, and ultrafiltration) produces higher protein concentrations with fewer antinutritional factors, dry fractionation is more sustainable and preserves protein functionality [2]. However, these processing operations must be carefully optimized to minimize the adverse effects of the antinutritional factors without affecting the desirable sensory attributes of the chickpeas [3]. Chickpeas are categorized into Kabuli and Desi genotypes, distinguished by size, shape, and color. In addition to being rich in macronutrients, their key micronutrients include calcium, phosphorus, magnesium, iron, and potassium [4]. Before being consumed or processed, chickpeas go through a variety of pre-processing treatments to improve their nutritional content, shorten processing times, and make them more palatable. They undergo a variety of physical, biochemical, or thermal treatments that enhance their digestibility and aid in the removal of antinutritional elements before being ingested in various ways. Cooking is a common practice to improve the protein quality of chickpeas by deactivating heat-sensitive antinutritional compounds. There has been growing interest in optimizing chickpea cooking methods in developed countries, where chickpeas are increasingly used as a replacement for animal-based foods to enhance nutritional quality. Chickpeas are highly nutritious, but their lengthy cooking times and the requirement for prolonged soaking before boiling are two key factors that restrict their consumer acceptability. Bad temperature and relative humidity conditions during the post-harvest storage of chickpeas can lead to various biochemical changes that cause pulses to become tough to cook or potentially degrade them in terms of both nutritional value and sensory qualities [5]. This creates the need to make them convenient by making them instant or ready to cook, which might benefit both food makers and consumers by encouraging the use of chickpeas. This reduces the requirement for soaking at the time of preparation and requires less time to rehydrate them during cooking. The freeze drying of pre-soaked chickpeas provides various benefits over traditional drying methods. Chickpeas are subjected to sub-zero temperatures under vacuum pressure, which removes water by sublimation, thereby reducing structural damage and preserving nutritional components. This can significantly reduce cooking time, lowering energy consumption and promoting more efficient cooking practices. Chickpeas can be made convenient to cook by first soaking and then drying them to formulate a shelf-stable product that can be rehydrated at the time of use. Biochemical changes and the associated quality deterioration that occur during the storage of chickpeas can be avoided.

This research explores how freeze-drying impacts cooking times and other properties of the chickpeas, including their overall functionality, antioxidant levels, how they behave when turned into a paste, and their structural characteristics. This comprehensive approach aims to fully understand the changes freeze drying brings to chickpeas.

## 2. Materials and Methods

Three cultivars of chickpeas, SH-1, BGD, and RV, were procured from the Pulse Research Station of SKUAST-K and belonged to the August 2022 harvest. Seeds were cleaned and stored at 20 °C until further use and were treated as control samples. The reagents/chemicals used in research were all analytical grade.

### 2.1. Preparation of Sample

About 200 g of each chickpea cultivar was soaked at a ratio of 1:3 containing 1 part sample and 3 parts deionized water for 24 h. The chickpeas were then lyophilized for 48 h in a vacuum flask at about 0.100 mBar and −55 °C in a benchtop freeze dryer (BUCHILaconic, Free Zone 4.5 Liter, NewCastle, WT, USA) to the moisture content of 10–12%. Some of the dried chickpeas were ground into powder and then sieved through a 75-mesh sieve to obtain flour of desired particle size for analysis. The flour and unground samples were packed in air-tight polythene bags for future analysis.

### 2.2. Composition of Seeds

Moisture (925.10), protein (920.87), fat (920.85), and ash (923.03), contents were evaluated according to standard methods. Total carbohydrates were calculated by the difference between the above components [6].

### 2.3. Engineering Properties of Seeds

#### 2.3.1. Length, Width, and Thickness of Seeds (mm)

Fifty random seeds were selected out from the lot of each freeze-dried and control chickpea cultivar to measure the length (L), width (W), and thickness (T) by using vernier callipers using the method given by [7].

#### 2.3.2. Diameter (mm)

The geometric mean diameter (GMD), arithmetic mean diameter (AMD), square mean diameter (SMD), and equivalent diameter (EQD) of the control and freeze-dried chickpea cultivars were evaluated by using following equations [7]:

GMD = (LWT)^1/3^

$$AMD=\frac{L+W+T}{3}$$

SMD = (LW + WT + TL)^1/2^

$$EQD=\frac{AMD+SMD+GMD}{3}$$

#### 2.3.3. Sphericity

The sphericity (ϕ) of samples was evaluated by using the following equation given by [8]:

$$\mathrm{Sphericity}\;(\phi )=\frac{\left(LWT\right)}{L}$$

#### 2.3.4. Volume and Surface Area

The volume (V) and surface area (S) of samples were determined using major grain measurements given by [9].

$$\mathrm{Volume}=\frac{\pi \left(GMD\right)L^{2}}{6(2L-GMD)}$$

$$\mathrm{Surface}\;\mathrm{area}=\frac{\pi \left(GMD\right)L^{2}}{2L-GMD}$$

#### 2.3.5. Shape Factor

The shape factor (λ) was calculated using seed volume and surface area of the samples given by [10].

$$\mathrm{Shape}\;\mathrm{factor}\;(\lambda )=\frac{a}{b}$$

where

$$a=\frac{v}{w}$$

$$b=\frac{s}{w}$$

#### 2.3.6. Thousand Kernal Mass

In order to calculate the thousand-grain mass (M1000), an electronic scale was used to weigh 100 randomly chosen grains, registering a measurement of 0.001 g. The result was then multiplied by 10 to obtain a 1000-seed basis.

#### 2.3.7. Bulk and True Density

The bulk and true density of the chickpea cultivars were evaluated using the method given by [11].

#### 2.3.8. Porosity

The porosity (ε) of the bulk is the ratio of spaces in the bulk to its bulk volume, and the porosity of seeds was determined by the following equation:

ε = 100 [1 − (ρ_b_/ρ_k_)]

where ε is the porosity in percentage, is the bulk density in g mL^−1^, and ρ_k_ is the seed density in g mL^−1^.

#### 2.3.9. Compressibility Index and Hausner Ratio

Using measured values for bulk density (ρb) and tapped density (ρt), the compressibility index (CI) and Hausner ratio (HR) were computed using the formula provided by [12].

CI = 100 × [(ρt − ρb)/ρt]

HR = ρt/ρb

### 2.4. Cooking Properties

#### 2.4.1. Cooking Time

The cooking time of the chickpea cultivars was evaluated using the method given by [13]. The cooking time was measured from the moment the seeds were added until the desired level of softness was reached, i.e., a seed can easily be pressed between the thumb and index finger.

#### 2.4.2. Gruel Solid Loss

For the shortest possible cooking time, 20 g of seeds was boiled in 200 mL of double-distilled water. After being moved to 500 mL beakers, the gruel was then heated at 110 °C until it was entirely dry. After that, the solids were weighed, and the percentage of gruel solid loss was calculated.

#### 2.4.3. Cooked Length/Breadth Ratio

After cooking for a minimal amount of time, the total length and width of ten seeds were measured. The length/breadth ratio was found by dividing the total length of ten cooked seeds by the cumulative breadth of the cooked seeds.

#### 2.4.4. Water Uptake Ratio of Cooked Seed

For the minimum cooking time, twenty grams of seeds was boiled in 200 mL of double-distilled water; they were then drained, and the surface water was removed from them using filter paper. Weighing the samples led us to determine the water uptake ratio, which is equal to the weight gained after cooking divided by the initial weight of the seeds before cooking.

### 2.5. Functional Properties

#### 2.5.1. Water and Oil Absorption Capacity

Flour samples of each freeze-dried and control sample (2.5 g on db.) were taken in pre-weighed tubes and were mixed with 20 mL of distilled water and 20 mL of oil (refined oil); they were then stirred for 30 min at 25 °C, and the mixture was centrifuged at 3000 rpm for 10 mL. The supernatant was decanted, and the gain in weight was then expressed as the oil–water absorption capacity as per [14] with slight modifications.

#### 2.5.2. Emulsion Capacity and Stability

The emulsion capacity and stability of the chickpea flour were determined as per the protocol given by [14], in which the samples (0.2 g) were taken on a dry basis in graduated centrifuge tubes, and 20 mL of distilled water and 20 mL of oil was added in the tubes containing the samples. The mixture was then homogenized to obtain the emulsion, and the tubes were then centrifuged at 2000 rpm for 5 min. The ratio of the height of the emulsion layer to the total height of the mixture was expressed as the emulsion capacity and was estimated according to the formula given below:

$$EC\;\%=\frac{Height\;of\;emulsion\;layer\;(\mathrm{cm})}{Total\;Height\;\;(\mathrm{cm})\;\;}\times 100$$

The emulsion stability of the flour samples was evaluated by using the formula given below after heating (80 °C) the tubes for 30 min.

$$ES\;\%=\frac{Height\;of\;emulsion\;layer\;after\;heating\;(\mathrm{cm})}{\;Height\;before\;heating\;(\mathrm{cm})\;\;}\times 100$$

#### 2.5.3. Foaming Capacity (FC) and Foaming Stability (FS)

The foaming capacity of the chickpea flour was evaluated as per the method given by [15], in which 2 g of each flour sample of the control and freeze-dried chickpeas was mixed with 100 mL of distilled water. The samples were thoroughly mixed by using a high-speed homogenizer (Wise Tis) for 1 min at 10,000 rpm. The foaming capacity of the flour samples was evaluated by the increase in volume of the flour mixture after homogenization given by the following formula:

$$FC\%=\frac{Volume\;after\;whipping-Volume\;before\;whipping}{Volume\;before\;whipping}\times 100$$

The foam stability of flour samples was evaluated by measuring the decrease in foam volume with time and determining the half-life as per the method given by [16].

$$FS\%=\frac{Foam\;volume\;after\;standing\;time\;(60\;min)}{Intial\;Foam\;Volume}\times 100$$

#### 2.5.4. Swelling and Solubility Indices

The swelling and solubility indices of the flour were determined using the method of [15]. Flour samples (0.2 g) from the control and freeze-dried chickpeas were taken in pre-weighed centrifuge tubes, and then 10 mL of distilled water was poured. The tubes were then incubated in a water bath at temperatures of 50, 60, 70, 80 and 90 °C for 30 min with intermittent vortexing after every 5 min. The tubes were then allowed to cool at room temperature and centrifuged at 4000× *g* for 10 min. The supernatant was then decanted into pre-weighed petri dishes, which were dried at 110 °C for 12 h. The gain in weight was expressed as the solubility index of the chickpea flour, and the gain in weight in the centrifuge tube was expressed as the swelling index of the flour.

### 2.6. Color

The surface color of the chickpea seeds of different cultivars of both the control and freeze-dried samples was evaluated using Ultra Scan VIS Hunter Lab (Hunter Associates Laboratory Inc., Reston, VA, USA) after being standardized by using Hunter Lab color standards and ‘L’ (lightness), ‘a’ (redness to greenness), and ‘b’ (yellowness to blueness) values.

### 2.7. Pasting Properties

The pasting properties of the flours were determined by using a rapid-visco analyzer (RVA Starch Master TM, Newport Scientific, Warriewood, Australia). The test profile STD1 (Newport scientific method 1, Version 5, 1997) was used for the determination of the pasting characteristics of the freeze-dried and control chickpea flour samples. The pasting curves obtained were analyzed using an RVA Starch Master Software Setup Tool (SMST) to obtain the characteristic parameters like peak viscosity, final viscosity, breakdown, and setback viscosity of the flour samples from both the control and freeze-dried chickpeas. An amount of 3 g (MC-12%) of flour of both the control and freeze-dried chickpeas was dispersed in distilled water (25 mL) and then stirred in RVA canisters. The pasting curves obtained were analyzed using an RVA Starch Master Software Setup Tool (SMST) to obtain the characteristic parameters like peak viscosity (PV), final viscosity (FV), breakdown (BD = PV − TV), and setback (SB = FV − TV).

### 2.8. Anti-Oxidant Activity

#### 2.8.1. Preparation of Extract

To prepare extracts of both the freeze-dried and control chickpea flour, 5 g of each cultivar of both the control and freeze-dried chickpea flour was mixed in 50 mL of methanol separately and was allowed to stir on magnetic stirrer for 72 h; after that, the mixture was filtered through Whatman filter paper, and the filtrate was used for analysis.

#### 2.8.2. DPPH (1, 1-Diphenyl-2-Picrylhydrazyl) Radical Scavenging Assay

The protocol followed for determining the DPPH radical scavenging activity was given by [17]. A 100 µL aliquot from the filtered extract was reacted with 3.9 mL of a 6 × 10^−5^ mol/L DPPH solution. The absorbance of samples was read for 0 and 30 min at 515 nm using methanol as a blank. It was determined using the following formula:

$$DPPH\;radical\;scavenging\;activity\%=\frac{A\;of\;control\;at\;0t-A\;of\;sampleat\;30t}{A\;of\;control\;at\;0t}\times 100$$

#### 2.8.3. Total Phenolic Content (TPC)

The procedure for determining the total phenolic content of the samples was given by [18]. The flour samples from both the control and freeze-dried chickpeas were taken (200 mg db) and mixed with 4 mL acidified methanol (HCl–methanol–distilled water mixed at a ratio of 1:80:10) for extraction; the mixture was then kept at room temperature (25 °C) for 2 h. The mixture was then centrifuged at 3000× *g* for 10 min. The supernatant obtained was then filtered, and the filtrate was collected. An aliquot (200 µL) from the extracted filtrate was taken, in which 1.5 mL of Folin’s reagent (10 × diluted) was added. The chemicals were then vortexed and allowed to equilibrate for 5 min. The mixture was then neutralized by mixing it with 1.5 mL of sodium carbonate (60 g/L). The samples were then incubated at room temperature for 90 min. Once the incubation was over, the absorbance of the samples was taken at 725 nm. A standard curve of gallic acid was prepared, and the TPC of the samples was expressed as gallic acid equivalents (GAEs) per gram of sample.

### 2.9. Texture Profile Analysis

Using a 50 kg load cell, a Texture Analyzer (Model XT2i; Stable Microsystems Ltd., Godalming, UK) was used to examine the texture of the cooked seeds. The heavy-duty platform of texture analyzer was used to set the seeds in their natural resting posture. A disc-type probe measuring 75 mm in diameter was used to execute the texture profile analysis (TPA) test for 50% compression levels at a test speed of 2.0 mm min^−1^. The TPA curve was used to calculate adhesiveness and hardness. Five determinations on average were reported.

### 2.10. FTIR

The FTIR spectra of the chickpea flour samples were recorded using an FTIR spectrometer system (Cary 630 FTIR, Agilent Technologies, Santa Clara, CA, USA). The analysis was carried out at room temperature, and spectra were acquired in the range of 4000–800 cm at a resolution of 2 cm^−1^ using Resolution Pro software version 2.5.5 (Agilent Technologies, USA).

### 2.11. Scanning Electron Microscopy

Using a scanning electron microscope (Gemini SEM-500, Jeol, Tokyo, Japan), the microstructure of both the control and freeze-dried chickpea flours was examined. After being coated with gold using a sputter coater, the flours were affixed to stubs using double-sided carbon tape and examined.

### 2.12. Statistical Analysis

The data reported in this study are averages of triplicate observations. An ANOVA was applied to determine the differences between the means using the commercial statistical package (SPSS, Inc., Chicago, IL, USA).

## 3. Results

### 3.1. Proximate Composition

The Proximate compositions of the different chickpea flour cultivars were evaluated, in which the carbohydrate content ranged between 60.791 and 67.882%: the carbohydrate content of RV and BGD varied insignificantly (≤0.05), and the carbohydrate content of SH-1 varied significantly compared to the others. The protein content of the cultivars ranged between 16.588 and 18.857%, and the protein content varied considerably among the cultivars. The fat content ranged from 3.183 to 3.610% and varied substantially within the cultivars. The ash content varied from 1.166 to 2.660%, and also varied significantly among the cultivars. The variations in the three types of legume flours’ genetic makeup, cultivars, and growth conditions can be attributed to the disparities in their chemical compositions. Similar results were seen in the study of different chickpea cultivars by [19].

#### 3.1.1. Engineering Parameters

The length (L), width (W), thickness (T), arithmetic mean diameter (AMD), geometric mean diameter (GMD), SMD, EQD, volume (V), surface area sphericity (s), aspect ratio, shape factor, M1000, bulk density (BD), tapped density (TD), porosity, Husner’s ratio (HR), and hydration capacity (HC) are presented in Table 1.

#### 3.1.2. Axial Dimensions

The main dimension L of the control chickpea cultivars was found in the range of 8.08–9.84 mm, and for the freeze-dried samples, it ranged between 9.57 and 10.96 mm. The L of the control samples increased significantly among the cultivars and with the freeze-dried chickpea cultivars. The B of the control chickpeas was between 6.28 and 8.53 mm and for the freeze-dried chickpeas was between 7.39 and 9.10 mm. The T of the control and freeze-dried chickpea cultivars was found in the range of 6.05–7.36 mm and 6.68–8.20 mm, respectively. The L, B, and T of the freeze-dried chickpeas was increased significantly due to the absorption of water while soaking, which resulted in the volumetric expansion of the seeds, thus causing the increase in dimensions. The values of the main dimensions varied significantly among the cultivars and with the freeze-dried samples.

#### 3.1.3. Diameter (mm)

The AMD, GMD, SMD, and ED of the control samples were in the range of 6.80–8.58, 6.70–8.33, 11.87–14.80, and 8.48–10.57 mm, respectively, and in the control samples, these ranged between 7.88 and 8.80, 7.62 and 8.94, 13.56 and 15.88, and 9.69 and 11.34 mm, respectively. The diameter values of the freeze-dried chickpeas increased more significantly than the control chickpeas because of the volumetric expansion or hydration of starch during soaking. The values varied considerably among the cultivars of both the control and freeze-dried chickpeas.

#### 3.1.4. Sphericity and Shape Factor

The control and freeze-dried chickpea sphericity values were in the range of 0.78–0.84 and 0.77–0.87, respectively. The sphericity of the FDBGD and FDSH (freeze-dried) samples was found to decrease, while in FDRV, the sphericity was found to increase. The sphericity values varied significantly among the cultivars of both the control and freeze-dried chickpeas and with each other. The shape factor of both the control and freeze-dried chickpeas was relatively the same. The values varied insignificantly in the range of 0.16–0.18 among the control and freeze-dried cultivars. The change in the sphericity and shape factor occurred mainly during soaking, as the soaking caused the changes in dimensions, which are related to these properties.

#### 3.1.5. Volume and Surface Area

The volume of the control and freeze-dried samples was in the range of 162.27–310.78 mm^3^ and 243.11–381.43 mm^3^, respectively. The volume of the control samples varied significantly among the cultivars, as did that of the freeze-dried chickpeas. The surface area of the control samples was found in the range of 145.28–223.59, and in the case of the freeze-dried chickpeas, it was found to be increased significantly in the range of 190.91–255.71 mm^3^. The freeze-dried samples showed an increase in volume and surface area due to the absorption of water when the seeds were soaked due to swelling.

#### 3.1.6. The 1000-Kernel Mass

In the control samples, the 1000-kernel mass ranged between 296.30 and 492.73 g, and for the freeze-dried samples, it significantly decreased to 284.9–441.86 g. The decrease in mass of the freeze-dried chickpeas was primarily due to the freeze-drying process, which resulted in the removal of bound water from the chickpeas, leaving behind a light and highly porous structure.

#### 3.1.7. Porosity

The porosity of the control chickpeas was found to be significantly lower than the freeze-dried chickpeas in the range of 3.11–3.65%; however, in the freeze-dried chickpeas, it ranged from 3.98 to 8.51%. The porosity of freeze-dried chickpeas was increased due to the removal of water during freezing, making the structure porous without collapsing the structure of the chickpeas.

#### 3.1.8. Bulk Density and Tapped Density

The bulk density and tapped density of the control chickpeas was found to be in the range of 0.79–1.63 and 0.82–1.69, respectively, and for the freeze-dried samples it significantly decreased, ranging from 0.43 to 0.45 and 0.45to 0.49, respectively, due to the increase in porosity in the seeds during freeze drying.

#### 3.1.9. Hausner’s Ratio

This ratio for the control chickpeas was 1.03, and for the freeze-dried chickpeas, it ranged from 1.04 to 1.09. This ratio varied significantly with the control and within the freeze-dried cultivars. This ratio was found to decrease due to a considerable decrease in bulk density, which overall increased the Husner’s ratio of the freeze-dried chickpeas.

The engineering properties discussed above showed similar results compared to the results shown by [20].

#### 3.1.10. Hydration Capacity

The hydration capacity of the control samples was found in the range of 0.36–0.63, and for the freeze-dried samples, it ranged from 0.52 to 0.76. The hydration capacity of the freeze-dried chickpeas was found to be relatively higher than control samples due to increased porosity because of freeze drying.

### 3.2. Cooking Properties

**Figure 1:**
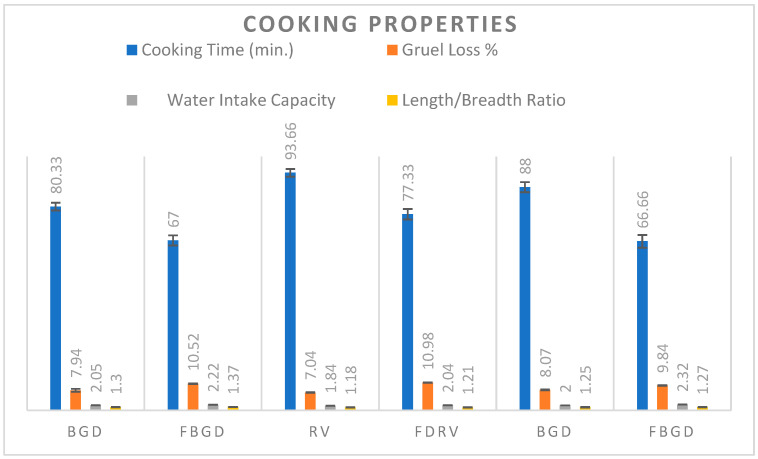
Cooking properties of control and freeze-dried chickpeas. Bar graph showing cooking properties of control and freeze-dried chickpeas varied significantly (*p* ≤ 0.05) with each other. Values expressed are mean ± standard deviation.

The cooking properties of various chickpea cultivars are given in Figure 1.

#### 3.2.1. Cooking Time

The cooking time of the control chickpea cultivars ranged between 80.33 and 93.66 min, and for the freeze-dried chickpeas, it decreased significantly in the range of 67.00–77.33 min. The cultivars varied significantly with each other, and variations were also found within the cultivars. Changes in cooking time among the cultivars might be due to the changes in seed cultivar, variety, growing location, production year, and genetics. However, the decreases in cooking time in the case of the freeze-dried chickpeas was due to freeze drying, as it eliminates water and leaves behind a porous, sponge-like structure [21]. During rehydration, the porous nature of the pulses makes it possible for water to enter fast, greatly accelerating the cooking process, as the pulses’ cellular structure is partially broken down during the freeze-drying process, cutting down on total cooking time. The results for cooking time in the control chickpeas were similar to the results of cooking time for various cultivars of chickpeas shown by [22].

#### 3.2.2. Gruel Loss

The gruel loss of the control chickpea cultivars during cooking was found in the range of 7.04–8.07%, which was in agreement with results shown by [23]. For the freeze-dried samples, it increased significantly in the range of 9.74–10.98%. The gruel loss percentage varied considerably among the chickpea cultivars. The increase in gruel loss percentage may be attributed due to the combined effect of both freeze drying and soaking, which makes the structure of chickpeas more porous and spongy. The cellular structure of pulses may sustain microdamage during the freeze-drying process. Cooking these pulses makes the damaged cells more likely to break down, which makes it possible for internal components to leak into the water more quickly. Thus, higher gruel loss during cooking is a result of structural alterations brought about by freeze drying, including cell damage, increased surface area, and altered starch behavior.

#### 3.2.3. Water Intake Capacity

The water intake capacity of the control samples ranged between 1.84 and 2.05, and in the freeze-dried chickpeas, it increased significantly in the range of 2.04–2.32. These results are comparable to the results shown by [22]. The water intake capacity of the control and freeze-dried chickpeas varied significantly among the cultivars and with each other; the water intake capacity is greatly affected in freeze-dried chickpea cultivars because the enhanced porosity by freeze drying facilitates faster and easier water penetration, hence augmenting the absorption capacity. A greater surface area is exposed to water as a result of the procedure. During cooking, this bigger area encourages more effective water absorption. The hydrophilic (attractive to water) soluble fibers, proteins, and carbohydrates found in freeze-dried food are retained/exposed [21].

#### 3.2.4. Length/Breath Ratio

The length/breadth ratio of the control chickpeas varied significantly in the range of 1.18–1.30, and in case of the freeze-dried samples, it increased significantly in the range of 1.21–1.30. The variations in the length/breadth ratio among the cultivars are attributed to genetic variations in the size of the chickpeas, and the increase in the length/breadth ratio in the freeze-dried chickpeas is mainly because of the combined impact of soaking and freeze drying, which increased porosity, thus increasing hydration, which overall increases the length/breadth ratio of the chickpeas.

### 3.3. Functional Properties

The functional properties of the control and freeze-dried chickpea flour is given in Table 2.

#### 3.3.1. Foaming Capacity and Stability

The foaming capacity and stability of the control and freeze-dried samples ranged between 42.58 and 45.16% and 44.37 and 47.20% and between 36.18 and 37.49% and 37.5 and 38.80%, respectively. The foaming capacity of the freeze-dried chickpea flour was found to be increased and varied significantly with the control chickpea flour, and the foaming stability of the freeze-dried flour was also found to be considerably increased in comparison to the control samples and among the cultivars. These results are comparable to the studies conducted by [24]. The soaking and freeze drying together enhanced the properties due to the increased extractability of proteins, as the freeze drying resulted in increased hydration due to increased porosity. It was reported that the molecular characteristics of proteins that are important for foaming include molecular rearrangement to prevent bubbles from becoming too close to the interface, segmental flexibility to facilitate unfolding at the interface, amphipathicity to enhance interfacial interactions, and solubility to enable rapid diffusion at the interface [25].

#### 3.3.2. Water and Oil Absorption Capacity

The water absorption capacity of the control and freeze-dried chickpea flours ranged between 0.84 and 0.98 g/g; similar results were found from the study conducted by [22]. For the freeze-dried flour, it ranged between 1.051 and 1.24 g/g. The results varied significantly with the control samples and within the cultivars, as the water absorption capacity of the freeze-dried samples was found to be increased. The oil absorption capacity of the control chickpea flour ranged between 0.73 and 0.98 g/g, and for the freeze-dried chickpea flour, it ranged between 0.909 and 980 g/g. The values for both the control and freeze-dried samples varied significantly within the cultivars, and the freeze-dried samples showed an increase in oil absorption capacity. The increase in both the properties, viz., water and oil absorption, in the freeze-dried chickpeas might be due to the porous structure that was formed during freeze-drying and the partial denaturation of proteins, which resulted in the exposure of more hydrophilic and hydrophobic sites that in turn enhanced starch–oil interactions.

#### 3.3.3. Emulsion Capacity and Emulsion Stability

The emulsion capacity of the control chickpea flour ranged between 42.35 and 44.84%, and for the freeze-dried samples, it varied from 46.59 to 54.49%. The values for the control and freeze-dried flour varied significantly with each other and within the cultivars. Protein denaturation, which increases the exposure of hydrophilic and hydrophobic groups, and the development of a porous structure, which boosts protein adsorption and stabilization at the oil–water interface, are the reasons why chickpea flour’s emulsion capacity rises after freeze drying. The emulsion stability of the control chickpea flour of the three cultivars ranged between 73.80 and 80.00% and for the freeze-dried flour emulsion stability ranged between 83.88 and 85.34%. The emulsion stability of the freeze-dried chickpea flour increased significantly due to improved protein functionality from denaturation, which enhances interfacial film formation, and the porous structure, which promotes better protein adsorption and stabilization and prevents phase separation over time. These results are comparable to the results of the study conducted on different chickpea cultivars by [26].

### 3.4. Pasting Properties

The pasting properties of the control and freeze-dried chickpea flour are given in Table 3. This property varies significantly among the cultivars and with the freeze-dried chickpea flour. PV ranged from 1064.33 to 1217.33 cP for the control samples, and for the freeze-dried samples, it ranged between 1288.66 and 1437.66 cP. The TV values for the control samples ranged between 874.33 and 944.33, and for the freeze-dried samples, it ranged from 1106.66 to 1173.66 cP. The peak and trough viscosities for the freeze-dried samples were increased. The freeze-dried chickpea flour’s peak and trough viscosities rose as a result of protein denaturation, which strengthens the viscous network, and improved starch gelatinization from the broken granular structure brought on by freeze drying, which improves water absorption and swelling. Peak viscosity is the point at which starch granules swell the most, which was shown to be higher in the freeze-dried flour than the control dry bean flour. The rate of granule swelling, amylose leaching, and the creation of amylose–lipid complexes are all indicated by trough viscosity. The breakdown and final viscosities varied significantly (≤0.05) in both the control and freeze-dried flour samples and among the cultivars, which ranged between 165.66 and 345.33 cP and 175.33 and 264.00 cP, respectively. The final viscosities for the control samples and freeze-dried samples ranged from 1025.33 to 1113.00 cP and 1367.66 to 1673.00 cP, respectively, and the setback viscosity of the control and freeze-dried chickpea flour ranged between 126.66 and 168.66 and 261.00–524.66 cP. Moreover, the pasting time of the control and freeze-dried samples ranged from 6.69 to 6.95 min and 6.13 to 6.63 min, respectively, and the pasting temperature ranged from 79.31 to 82.63 °C and 76.32 to 82.63 °C. The pasting time and temperature of the freeze-dried samples were found to be decreased because of the samples’ increased porosity and better water absorption, respectively. Freeze-dried dry bean flour has lower pasting temperatures and times than traditionally dried flour. These factors improve the efficiency of starch swelling and gelatinization. The results for the control samples are similar to the results of the study conducted on chickpea cultivars by [23].

### 3.5. Swelling and Solubility Indices

The swelling and solubility indices of the chickpea flour varied significantly (≤0.05) among the cultivars and with each other throughout the temperature range of 50–90 °C, as presented in Table 4. It was found that both the swelling and solubility indices of the control and freeze-dried samples increased with the increase in temperature in the range of 1.633–6.683, 1.75–6.683, and 1.433–22-7.31 for BGD, RV, and SH, respectively, and for the freeze-dried samples it ranged from 2.123 to 7.11, 2.436 to 7.28, and 2.823 to 7.56 for FDBGD, FDRV, and FDSH, respectively. The swelling index of the freeze-dried flour was found to be increased significantly compared to the control samples. The solubility index of the samples also increased considerably (≤0.05) throughout the temperature range of 50–70 °C and then decreased in the temperature range of 70–90 °C among cultivars and with each other. The solubility index of the control chickpea flour ranged from 0.300 to 0.446, 0.201 to 0.351, and 0.241 to 0.413 for BDG, RV, and SH, respectively, and for the freeze-dried chickpea flour, it ranged from 0.422 to 0.492, 0.297 to 0.410, and 0.314 to 0.471 for FDBGD, FDRV, and FDSH, respectively. The swelling and solubility indices of the freeze-dried chickpea flour were found to be higher than for the control flour. This may be attributed to the increased water-holding capacity and increased porosity in freeze-dried flour and the gelatinization of starch, in which the amylopectin fraction plays a significant role. Similar results were observed in study conducted by [27].

### 3.6. Color

**Figure 2:**
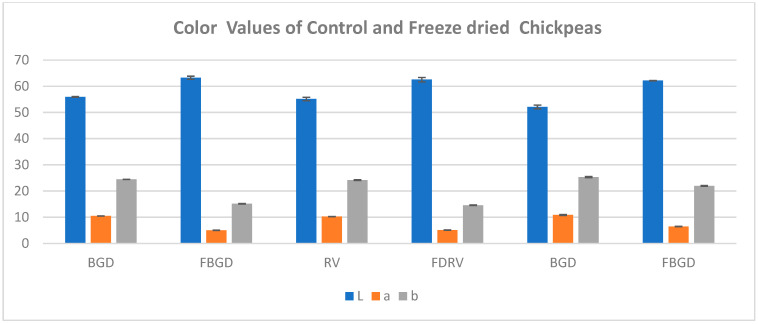
Color values of control and freeze-dried chickpeas, which varied significantly (*p* ≤ 0.05). Values expressed are mean ± standard deviation.

The color parameters of the control chickpea cultivars varied significantly (≤0.05) among the cultivars and with the freeze-dried chickpeas, as presented in Figure 2. The “L” value ranged between 52.12 and 55.94 for the control samples, and for the freeze-dried chickpeas, the L value ranged between 62.166 and 63.26, which was significantly higher than that found for the control chickpeas because of the prior soaking, which resulted in the removal of phenolics present in the seed coat during soaking. The “a” value of the control samples ranged between 10.26 and 10.87 for the control samples, and for the freeze-dried samples it ranged between 5.02 and 6.46, which was found to be lower than the control chickpeas and indicated a lighter red color in the freeze-dried than control samples. The b value was found to be in the range of 24.18–25.323 in the control chickpeas, and for the freeze-dried chickpeas, it was significantly lower than the control samples, ranging between 14.596 and 21.933, which indicates that the control samples were more yellow than the freeze-dried chickpeas. Lower “a” & “b” values of the freeze-dried samples were found because of the prior soaking, which resulted in the removal of water-soluble carotenoids present in the chickpeas during soaking [28]. The results showed that the freeze-dried samples were more lighter in yellow color than the control chickpeas, and the results are comparable to the study conducted by [22].

### 3.7. Antioxidant Activity

**Figure 3:**
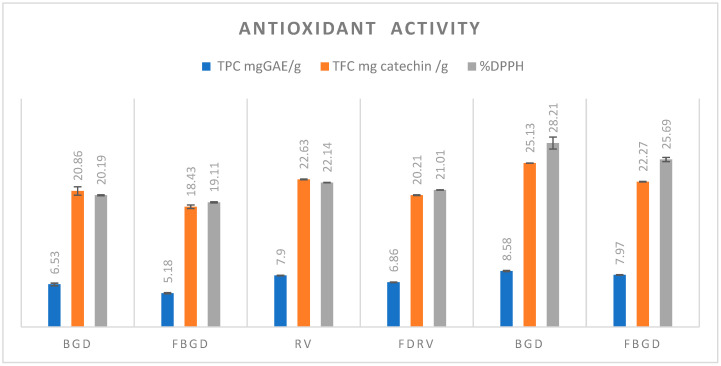
Antioxidant activity of control and freeze-dried chickpea flours of different cultivars, which varied significantly (*p* ≤ 0.05). Values expressed are mean ± standard deviation.

The antioxidant activity of the control and freeze-dried chickpea flours is given in Figure 3. The DPPH scavenging activity of the control samples varied significantly among the cultivars, and with the freeze-dried chickpea flour, it varied between 20.19 and 28.21%. For the freeze-dried flour, it also varied considerably among the cultivars and ranged between 19.11 and 25.69%. The DPPH radical scavenging activity for the freeze-dried chickpea flour significantly decreased because of the prior soaking, which resulted in the leaching of antioxidant components that are mostly concentrated on the seed coat [29]. Because of this, the TPC and TFC of the control samples also decreased. The TPC ranged between 6.53 and 8.58 mg/gGAE in the control samples and decreased in the case of the freeze-dried dry bean flour between 5.18 and 7.97 mg/gGAE. The results for the control and freeze-dried samples varied significantly (≤0.05) among the cultivars and with each other, and the control chickpea flour varied significantly (≤0.05). The total flavonoid content among the cultivars of the control samples ranged between 20.86 and 25.13 (mg catechin/g), and for the freeze-dried flour, it varied significantly among the cultivars between 18.43 and 23.27 (mg catechin/g). The results are comparable to the study conducted by [30].

### 3.8. Textural Properties

The textural properties of the control and freeze-dried chickpeas are given in Table 5. The hardness of the cooked control chickpeas was found in the range of 2.72–3.91 kg, and for the freeze-dried chickpeas, it reduced significantly in the range of 1.48–2.05 kg. Because of variations in the structure and seed coat that occur during drying, cooked freeze-dried chickpeas have a lower hardness than traditionally dried ones. Freeze-drying causes little harm to the seed coat while maintaining the cellular structure and producing an open and porous texture. As a result, chickpeas become softer during cooking because water can more readily pass into the seed. Conventional drying, however, breaks down cell walls, compacts the structure, and solidifies the seed coat, which results in a slower rate of water absorption and a tougher cooked chickpea. The adhesiveness of the control samples ranged from 0.24 to 0.38 kg*s, and for the freeze-dried samples, it increased significantly in the range of 0.47 to 0.55 kg. The sublimation, concentration, and exposure of soluble hydrophilic components such as starch, proteins, and sugars, as well as the enhanced water-binding ability of denatured proteins and disrupted starch granules, are the primary causes of the increased adhesiveness of freeze-dried chickpeas. The cohesiveness of the cooked control chickpeas was found in the range of 0.15–0.22, and for the cooked freeze-dried chickpeas, it reduced significantly in the range of 0.12–0.18 due to structural weakening due to porosity and brittleness from freeze-drying, protein denaturation, which reduces network formation, uneven rehydration and rapid water uptake causing structural collapse, and the loss of strong interactions between proteins and starch. The chewiness of the control samples was found in the range of 2.62–3.76, and for the freeze-dried samples, it reduced significantly in the range of 1.42–2.18. The textural properties were comparable to studies conducted by [22].

### 3.9. ATR-FTIR Spectroscope Analysis

FTIR spectroscopy is a widely used technique used to identify the functional groups and structural changes in several food products. In this study, it was used to observe the structural differences among different cultivars of chickpea flours. Supplementary Figure S1 shows several important peaks in the FTIR spectrum, which can help to identify the proteins, fats, and carbohydrates present in the flours. The first peak that can be detected is at the range of 1000–1100 cm^−1^, which is a typical peak for polysaccharides, and this peak indicates the coupling of the C=O or the C=C stretching modes [31]. Furthermore, the intensity of this peak suggests a relative estimation of the polysaccharide content in a system, and the highest intensity belonged to this peak, indicating that the flours consist of mostly polysaccharides. The peak observed around 1700 cm^−1^ provides information about the C=O stretching modes of fats. Moreover, Amide I (~1600 cm^−1^), Amide II (~1500 cm^−1^), and Amide A (~3300 cm^−1^) bands, where C=O, C=N, and N=H stretching modes of proteins are observed, can be easily detected from the FTIR spectrum [31]. Overall, these peaks provide information regarding the polysaccharides, fats, and proteins in the flours. From the FTIR spectrum, no change in the chemical composition was observed; however, a change in intensities was seen in the region of 3000–3600 cm^−1^ in the freeze-dried chickpea flours because of the removal of water (OH) during freeze drying. The results obtained for the control samples are in accordance with the results shown by [32].

### 3.10. Scanning Electron Microscopy

The SEM images of the control and freeze-dried chickpea flours are shown in Supplementary Figure S2. The control flour samples examined under a scanning electron microscope (SEM) showed elliptical or oval granules. The irregularities observed in the SEM images of the flour particles might be caused by pieces of the protein matrix or associated protein entities that broke off during grinding. Another possible reason might be the linked fiber and mineral components, as [33] studied. This shape resembled that of the flour from several chickpea varieties as shown by SEM pictures in the research conducted by [34]. The structure of the control chickpea flour seems to be denser and more substantial. Less cellular wall damage appears to be the result of the beans not having undergone any extensive processing, such as soaking or freeze drying. The micrographs of the control samples are comparable to the results shown by [35]. The structure of the soaked and freeze-dried chickpea flour, on the other hand, is significantly more fractured and disjointed, as the freezing resulted in the formation of crystals, which in turn created pressure in the particles, resulting in distortion of the structure. The process of freeze drying tends to produce more fractures, which is why the cellular structure collapsed or cracked. In comparison to the control chickpea flour, the particles are much smaller and more asymmetrical in form. Water is absorbed into the chickpeas during the soaking process, which causes the cell walls to expand and shatter when they freeze and sublimate (freeze drying). Significant structural damage is indicated by the existence of these gaps and smaller, more fragmented particles. Similar properties of freeze-dried chickpeas were revealed in the study conducted by [5].

## 4. Conclusions

The results of this study show that different chickpea cultivars’ cooking, functional, pasting, and structural qualities are greatly improved by freeze drying. The biggest advantage was the noticeable decrease in cooking time, which established freeze-dried chickpeas as a quicker choice for food processors and customers. Furthermore, improvements in the pasting behavior, swelling index, and water absorption capacity highlight how freeze drying might increase chickpeas’ utility in a variety of culinary applications. The observed decrease in antioxidant activity, however, suggests that although freeze drying successfully enhances functional and physical characteristics, it may jeopardize some nutritional aspects. Future studies should concentrate on creating plans to maintain or raise antioxidant levels before, during, or after the freeze-drying procedure. All things considered, freeze-drying shows potential as a method of preserving chickpeas, especially for improving their functional performance and cooking efficiency. It may be used in both home and commercial food systems.

## Figures and Tables

**Table 1:** Physical and engineering parameters of control and freeze-dried chickpea cultivars (*n* = 3).

Cultivars	L (mm)	B (mm)	T (mm)	AMD (mm)	GMD (mm)	SMD (mm)	ED (mm)	Sphericity	V (mm^3^)	SA	SF	BD	TD	HR	P%	M (1000) g	HC
BGD	8.76 ± 0.67 ^a^	6.28 ± 0.32 ^a^	6.23 ± 0.22 ^a^	7.09 ± 0.41 ^a^	6.86 ± 0.36 ^a^	12.19 ± 0.66 ^a^	8.71 ± 0.47 ^a^	0.78 ± 0.54 ^ab^	177.51 ± 0.03 ^a^	155.18 ± 0.53 ^a^	0.18 ± 0.18 ^b^	1.62 ± 0.02 ^d^	1.68 ± 0.01 ^d^	1.03 ± 1.24 ^a^	3.27 ± 1.24 ^a^	390.33 ± 13.23 ^b^	0.36 ± 0.02 ^a^
FDBGD	10.96 ± 0.22 ^c^	8.11 ± 0.91 ^cd^	7.33 ± 0.13 ^b^	8.80 ± 0.42 ^cd^	8.48 ± 0.30 ^cd^	15.12 ± 0.59 ^cd^	10.80 ± 0.43 ^cd^	0.77 ± 1.35 ^a^	337.02 ± 0.01 ^cd^	238.28 ± 0.32 ^cd^	0.17 ± 0.05 ^ab^	0.45 ± 0.00 ^ab^	0.49 ± 0.00 ^b^	1.09 ± 0.00 ^c^	8.51 ± 0.00 ^c^	386.26 ± 1.20 ^b^	0.59 ± 0.58 ^d^
RV	9.84 ± 0.46 ^b^	8.53 ± 0.87 ^d^	7.36 ± 0.44 ^b^	8.58 ± 0.54 ^c^	8.33 ± 0.56 ^c^	14.80 ± 1.00 ^c^	10.57 ± 0.72 ^c^	0.84 ± 1.21 ^bc^	310.78 ± 0.10 ^c^	223.59 ± 1.06 ^c^	0.16 ± 0.10 ^a^	1.63 ± 0.00 ^d^	1.69 ± 0.00 ^d^	1.03 ± 0.00 ^a^	3.11 ± 0.35 ^a^	492.73 ± 17.60 ^d^	0.63 ± 0.01 ^e^
FDRV	10.26 ± 0.20 ^bc^	9.10 ± 0.52 ^d^	8.20 ± 0.17 ^c^	9.18 ± 0.30 ^d^	8.94 ± 0.27 ^d^	15.88 ± 0.48 ^d^	11.34 ± 0.35 ^d^	0.87 ± 1.30 ^c^	381.43 ± 0.01 ^d^	255.71 ± 0.25 ^d^	0.16 ± 0.00 ^a^	0.45 ± 0.00 ^b^	0.49 ± 0.00 ^b^	1.07 ± 0.00 ^b^	7.28 ± 0.92 ^b^	441.86 ± 1.71 ^c^	0.76 ± 0.01 ^f^
SH	8.08 ± 0.43 ^a^	6.51 ± 0.24 ^ab^	6.05 ± 0.52 ^a^	6.88 ± 0.40 ^a^	6.70 ± 0.38 ^a^	11.87 ± 0.67 ^a^	8.48 ± 0.48 ^a^	0.82 ± 0.90 ^abc^	162.27 ± 0.03 ^a^	145.28 ± 0.47 ^a^	0.17 ± 0.25 ^ab^	0.79 ± 0.00 ^c^	0.82 ± 0.00 ^c^	1.03 ± 0.00 ^a^	3.65 ± 0.02 ^a^	296.30 ± 12.4 ^a^	0.42 ± 0.02 ^b^
FDSH	9.57 ± 0.27 ^b^	7.39 ± 0.22 ^bc^	6.68 ± 0.57 ^ab^	7.88 ± 0.24 ^b^	7.62 ± 0.27 ^b^	13.56 ± 0.46 ^b^	9.69 ± 0.32 ^b^	0.79 ± 0.03 ^ab^	243.11 ± 23.42 ^b^	190.91 ± 11.40 ^b^	0.17 ± 0.00 ^ab^	0.43 ± 0.00 ^a^	0.45 ± 0.00 ^a^	1.04 ± 0.00 ^a^	3.98 ± 0.01 ^a^	284.9 ± 0.65 ^a^	0.52 ± 0.01 ^c^

L—length; B—breadth; T—thickness; AMD—arithmetic mean diameter; GMD—geometric mean diameter; SMD—squared mean diameter; ED—equivalent diameter; V—volume; SA—surface area; SF—surface factor; BD—bulk density; TD—tapped density; HR—Hausner’s ratio; P—porosity mass; HC—hydration capacity. Means in the rows and columns with different superscripts are significantly different at *p* ≤ 0.05. Values expressed are mean ± standard deviation.

**Table 2:** Functional properties of control and freeze-dried chickpeas (*n* = 3).

Cultivars	Foaming Capacity (%)	Foaming Stability (%)	Water Absorption Capacity (g/g)	Oil Absorption Capacity (g/g)	Emulsion Capacity (%)	Emulsion Stability (%)
BGD	42.58 ± 0.119 ^a^	36.18 ± 0.07 ^a^	0.97 ± 0.03 ^ab^	0.73 ± 0.02 ^a^	42.35 ± 1.206 ^a^	74.31 ± 1.07 ^c^
FD BGD	44.37 ± 0.05 ^b^	37.58 ± 0.55 ^b^	1.24 ± 0.01 ^c^	0.91 ± 0.02 ^bc^	46.59± 0.60 ^b^	84.91 ± 0.14 ^a^
RV	45.16 ± 0.04 ^c^	37.49 ± 0.62 ^b^	0.98 ± 0.02 ^ab^	0.97± 0.04 ^d^	44.84 ± 0.69 ^b^	80.00 ± 0.00 ^b^
FD RV	47.20 ± 0.12 ^d^	38.80 ± 0.14 ^c^	1.19 ± 0.00 ^c^	0.98 ± 0.01 ^bc^	50.69 ± 1.58 ^c^	83.88 ± 0.50 ^c^
SH	42.58 ± 0.119 ^a^	36.79 ± 0.04 ^a^	0.84 ± 0.18 ^a^	0.85± 0.05 ^b^	42.58 ± 0.12 ^b^	73.80 ± 3.07 ^a^
FDSH	46.76 ± 0.67 ^d^	37.62 ± 0.24 ^b^	1.05 ± 0.02 ^b^	0.96 ± 0.01 ^cd^	54.49 ± 1.62 ^d^	85.34 ± 0.11 ^c^

Means in the rows with different superscripts are significantly different at *p* ≤ 0.05. Values expressed are mean ± standard deviation.

**Table 3:** Pasting properties of control and freeze-dried chickpea (*n* = 3).

Cultivars	Peak Viscosity (cP)	Trough Viscosity (cP)	Breakdown Viscosity (cP)	Final Viscosity (cP)	Setback Viscosity (cP)	Pasting Time. (min.)	Pasting Temperature (°C)
BGD	1178 ± 49.51 ^b^	944.33 ± 37.16 ^abc^	235 ± 71.58 ^a^	1113± 43.31 ^a^	168.66 ± 8.08 ^b^	6.95 ± 0.075 ^d^	79.31 ± 1.23 ^ab^
FDBGD	1382 ± 21.00 ^d^	1128.66 ± 7.50 ^cd^	253.66 ± 13.50 ^a^	1622 ± 35.51 ^c^	493.00 ± 28.00 ^d^	6.23 ± 0.10 ^a^	79.9 ± 0.05 ^ab^
RV	1064.33 ± 9.29 ^a^	898.66 ± 3.51 ^ab^	165.66 ± 12.66 ^a^	1025.33 ± 4.04 ^a^	126.66 ± 3.05 ^a^	6.86 ± 0.06 ^cd^	80.8 ± 0.00 ^ab^
FDRV	1437.66 ± 6.50 ^e^	1173.66 ± 12.50 ^d^	264.00 ± 6.00 ^a^	1673 ± 24.00 ^c^	524.66 ± 11.50 ^d^	6.14 ± 0.14 ^a^	79.93 ± 0.05 ^ab^
SH	1217.33 ± 15.5 ^b^	874.33 ± 0.71 ^a^	345.33 ± 19.79 ^a^	1034.33 ± 10.60 ^a^	160.00 ± 9.89 ^ab^	6.69 ± 0.18 ^bc^	82.63 ± 0.56 ^b^
FDSH	1288.66 ± 23.50 ^c^	1106.66 ± 0.58 ^bcd^	175.33 ± 15.14 ^a^	1367.66 ± 2.51 ^b^	261.00 ± 2.00 ^c^	6.63 ± 0.04 ^b^	76.32 ± 0.35 ^a^

Means in the rows and columns with different superscripts are significantly different at *p* ≤ 0.05. Values expressed are mean ± standard deviation.

**Table 4:** Swelling and solubility indices of different cultivars of control and freeze-dried chickpea flours (*n* = 3).

Swelling Index	BGD	RV	SH	FDBGD	FDRV	FDSH
50 °C	1.63 ± 0.10 ^b^	1.75 ± 0.1 ^b^	1.43 ± 0.15 ^a^	2.12 ± 0.07 ^c^	2.44 ± 0.14 ^d^	2.82 ± 0.06 ^e^
60 °C	2.08 ± 0.16 ^a^	2.00 ± 0.13 ^a^	2.10 ± 0.08 ^a^	2.76 ± 0.03 ^b^	2.96 ± 0.05 ^c^	3.36 ± 0.04 ^d^
70 °C	4.35 ± 0.81 ^a^	4.38 ± 0.81 ^a^	3.93 ± 0.07 ^a^	4.56 ± 0.05 ^a^	5.79 ± 0.74 ^b^	4.27 ± 0.02 ^a^
80 °C	5.23 ± 0.26 ^a^	5.78 ± 0.25 ^b^	5.33 ± 0.10 ^a^	5.83 ± 0.87 ^b^	5.71 ± 0.22 ^b^	5.94 ± 0.03 ^b^
90 °C	6.68 ± 0.20 ^a^	6.68 ± 0.17 ^a^	7.31 ± 0.55 ^b^	7.11 ± 0.11 ^ab^	7.28 ± 0.05 ^b^	7.56 ± 0.06 ^b^
Solubility Index						
50 °C	0.30 ± 0.03 ^c^	0.20 ± 0.02 ^a^	0.24 ± 0.03 ^b^	0.42 ± 0.01 ^d^	0.29 ± 0.01 ^c^	0.31 ± 0.04 ^c^
60 °C	0.36 ± 0.04 ^bc^	0.30 ± 0.22 ^a^	0.32 ± 0.05 ^ab^	0.47 ± 0.02 ^a^	0.36 ± 0.01 ^bc^	0.38 ± 0.01 ^c^
70 °C	0.45 ± 0.02 ^c^	0.37 ± 0.02 ^a^	0.37 ± 0.02 ^a^	0.48 ± 0.01 ^d^	0.41 ± 0.01 ^b^	0.44 ± 0.02 ^c^
80 °C	0.32 ± 0.00 ^b^	0.27 ± 0.00 ^a^	0.32 ± 0.01 ^b^	0.42 ± 0.01 ^e^	0.39 ± 0.00 ^c^	0.41 ± 0.03 ^d^
90 °C	0.34 ± 0.03 ^a^	0.35 ± 0.011 ^a^	0.41 ± 0.01 ^b^	0.49 ± 0.03 ^c^	0.41 ± 0.01 ^b^	0.47 ± 0.05 ^c^

Means in the row & columns with different superscript are significantly different at *p* ≤ 0.05. Values expressed are mean ± Standard deviation.

**Table 5:** Textural properties of control and freeze-dried chickpea cultivars (*n* = 3).

Cultivars	Hardness (kg)	Adhesiveness (kg/s)	Cohesiveness	Chewiness
BGD	3.91 ± 0.82 ^d^	0.24 ± 0.04 ^a^	0.22 ± 0.06 ^b^	2.62 ± 0.24 ^bc^
FDBGD	2.05 ± 0.04 ^ab^	0.55 ± 0.02 ^d^	0.18 ± 0.05 ^ab^	2.18 ± 0.11 ^ab^
RV	3.33 ± 0.36 ^c^	0.38 ± 0.10 ^bc^	0.15 ± 0.03 ^a^	3.76 ± 1.16 ^d^
FDRV	1.99 ± 0.03 ^a^	0.47 ± 0.01 ^cd^	0.12 ± 0.01 ^a^	2.16 ± 0.04 ^a^
SH	2.72 ± 0.39 ^bc^	0.31 ± 0.01 ^ab^	0.18 ± 0.01 ^ab^	3.40 ± 0.46 ^cd^
FDSH	1.48 ± 0.11 ^ab^	0.47 ± 0.06 ^cd^	0.14 ± 0.01 ^a^	1.42 ± 0.25 ^ab^

Means in the rows and columns with different superscripts are significantly different at *p* ≤ 0.05. Values expressed are mean ± standard deviation.

## Data Availability

The original contributions presented in this study are included in the article/Supplementary Materials. Further inquiries can be directed to the corresponding author.

## Supplementary Materials

The following supporting information can be downloaded at: https://www.mdpi.com/article/10.3390/foods14101687/s1, Figure S1: FTIR graphs of control and freeze-dried chickpea flour; Figure S2: SEM micrographs of control chickpea flour and magnifications: (A) SH (20.00KX); (C) RV (20.00KX); (E) BGD (20.00KX). Freeze-dried chickpea flour cultivars: (B) FSH (10.00KX); (D) FRV (20.00KX); (F) FBGD(20.00KX).
