# Supplementary material for: Effect of Freeze-Drying on the Engineering, Cooking, and Functional Properties of Chickpea Cultivars Grown in an Indian Temperate Climate
Source: Foods. 2025 May 10;14(10):1687. doi: 10.3390/foods14101687 (PMC12111726; doi:10.3390/foods14101687)
Supplement: Supplementary file 1 [file foods-14-01687-s001.zip › foods-3619340-supplementary.pdf]

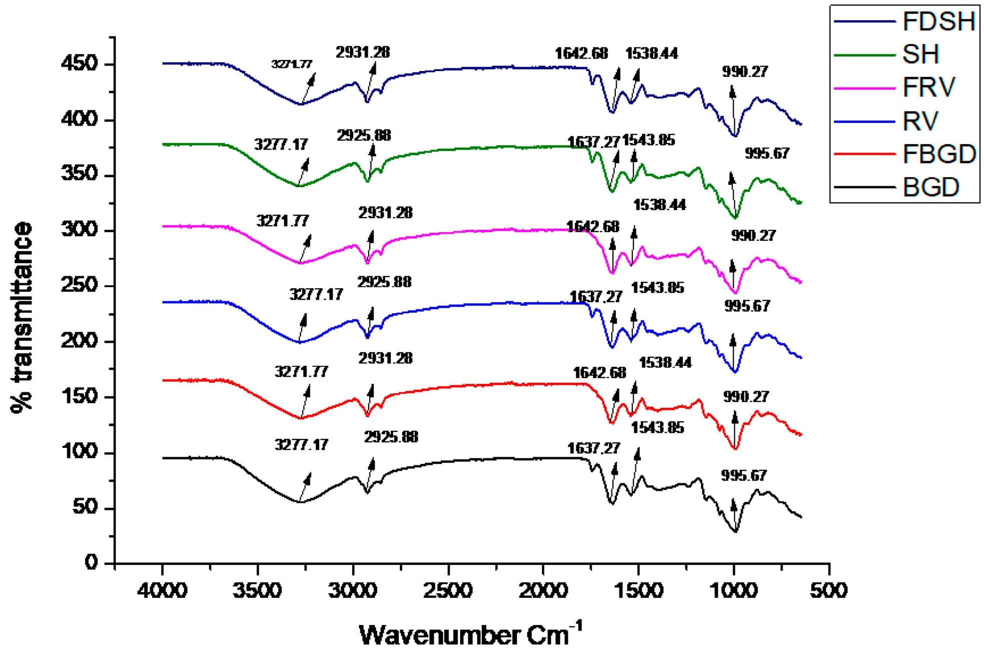

Supplementary Figure S1: FTIR graphs of control and freeze-dried chickpea flour.

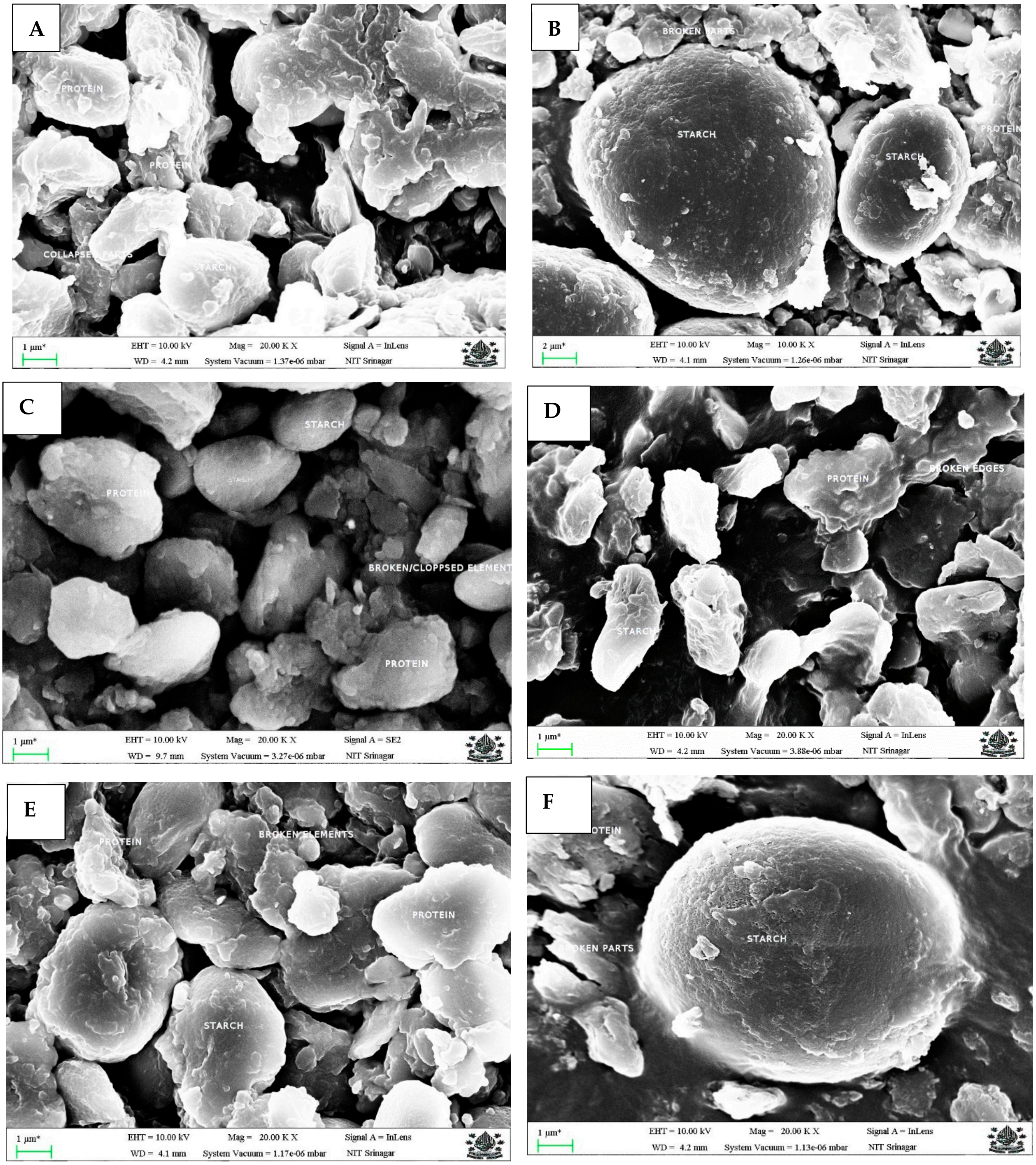

Supplementary Figure S2. SEM micrographs of control chickpea flour and magnifications: (A) SH (20.00KX); (C) RV (20.00KX); (E) BGD (20.00KX). Freeze-dried chickpea flour cultivars: (B) FSH (10.00KX); (D) FRV (20.00KX); (F) FBGD(20.00KX).
